# Supplementary material for: Aquaporin-7-Mediated Glycerol Permeability Is Linked to Human Sperm Motility in Asthenozoospermia and during Sperm Capacitation
Source: Cells. 2023 Aug 5;12(15):2003. doi: 10.3390/cells12152003 (PMC10416866; doi:10.3390/cells12152003)
Supplement: Supplementary file 1 [file cells-12-02003-s001.zip › cells-2509515-supplementary.pdf]

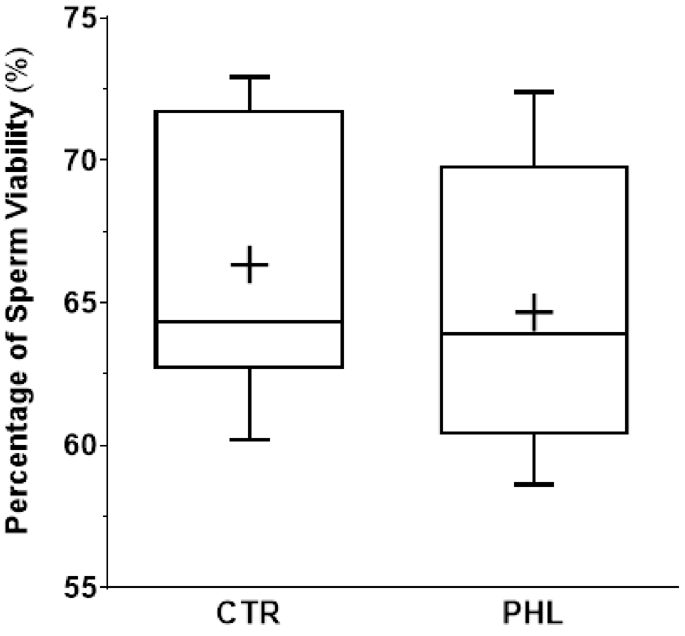

Figure S1: Percentage of sperm viability after incubation of isosmotic medium with and without phloretin (PHL) for 10 minutes. N=12

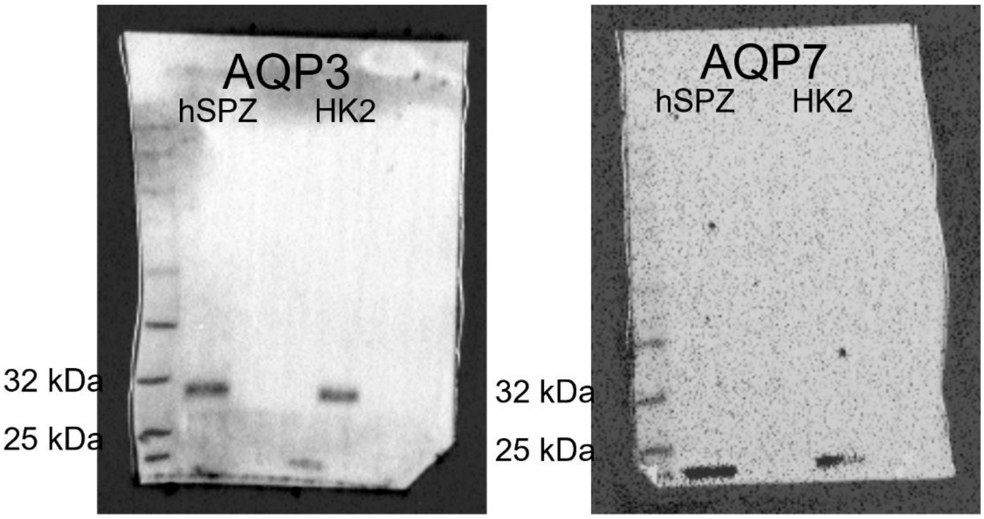

Figure S2: Representative blots of immunodetection of AQP3 and AQP7 in human spermatozoa (hSPZ) and human kidney cell line (HK2) for antibody specificity validation.
